# Selective Oxidation of HMF via Catalytic and Photocatalytic Processes Using Metal-Supported Catalysts

**DOI:** 10.3390/molecules23112792

**Published:** 2018-10-27

**Authors:** Alice Lolli, Valeriia Maslova, Danilo Bonincontro, Francesco Basile, Simona Ortelli, Stefania Albonetti

**Affiliations:** 1 Department of Industrial Chemistry “Toso Montanari”, Bologna University, Viale Risorgimento 4, 40136 Bologna, Italy; alice.lolli4@unibo.it (A.L.); valeriia.maslova2@unibo.it (V.M.); danilo.bonincontro2@unibo.it (D.B.); f.basile@unibo.it (F.B.); 2 C2P2, UMR 5265, CNRS–Univeristé de Lyon1 UCBL–CPE Lyon, Université de Lyon, 43 Boulevard du 11 Novembre 1918, 69616 Villeurbanne, France; 3 ISTEC-CNR, Institute of Science and Technology for Ceramics, National Research Council, Via Granarolo 64, 48018 Faenza, Italy; simona.ortelli@istec.cnr.it

**Keywords:** 5-hydroxymethyl furfural, spray-freeze drying, photocatalysis, TiO_2_, microemulsion

## Abstract

In this study, 5-hydroxymethylfurfural (HMF) oxidation was carried out via both the catalytic and the photocatalytic approach. Special attention was devoted to the preparation of the TiO_2_-based catalysts, since this oxide has been widely used for catalytic and photocatalytic application in alcohol oxidation reactions. Thus, in the catalytic process, the colloidal heterocoagulation of very stable sols, followed by the spray-freeze-drying (SFD) approach, was successfully applied for the preparation of nanostructured porous TiO_2_-SiO_2_ mixed-oxides with high surface areas. The versatility of the process made it possible to encapsulate Pt particles and use this material in the liquid-phase oxidation of HMF. The photocatalytic activity of a commercial titania and a homemade oxide prepared with the microemulsion technique was then compared. The influence of gold, base addition, and oxygen content on product distribution in the photocatalytic process was evaluated.

## 1. Introduction

**Scheme 1:**
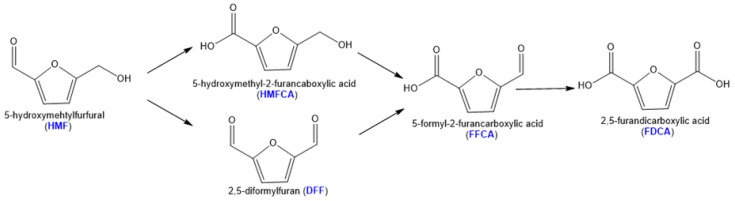
Reaction pathways for 5-hydroxymethylfurfural (HMF) oxidation.

5-hydroxymethyl furfural (HMF) is still one of the most-studied platform molecules for the production of fuels and chemicals from renewable biomass sources. This is thanks to its chemical structure, which includes a furan ring, a hydroxyl group, and a formyl group that can undergo different reactions such as reduction, oxidation, and esterification. HMF oxidation has been widely studied over the past two decades using different reaction conditions and catalysts [1,2,3,4,5]. Scheme 1 shows the general HMF oxidation pattern. Indeed, HMF can be transformed in different ways: the carbonyl group can be oxidized to the carboxylic moiety, producing 5-hydroxymethyl-2-furancarboxylic acid (HMFCA); the oxidation of the hydroxymethyl group can then produce FDCA via 5-formyl-2-furancaboxylic acid (FFCA) intermediate formation. Moreover, the formation of 2,5-diformylfuran (DFF) can also be observed, mainly in the absence of an added base and with metals other than Au [6,7,8].

Some HMF derivatives characterised by diols or diacid double functionalities are used in the polymer industry for bio polyester production [9]. As an example, 2,5-furandicarboxylic acid (FDCA), produced from HMF oxidation, is one of the most promising intermediates for the production of poly(ethylene furanoate) (PEF), the furan-based analogue to poly(ethylene terephthalate) (PET), which is the dominant polymer in beverage packaging industries. Recent results have demonstrated that poly(ethylene furanoate), when manufactured with bio-sourced ethylene glycol, provides a 100% renewable polymer with an enhanced oxygen and carbon dioxide permeability compared to PET, despite the fact that an increase in CO_2_ solubility is observed for PEF polymer [10,11]. Moreover, the chain mobility, reduced due to the suppression of the furan ring-flipping—because of an increased furan ring hindrance, which is characteristic for the bio-based polymer—affects the overall CO_2_ transportation properties. A decrease of the oxygen permeability and higher glass transition temperature are present in PEF with respect to its terephtalic acid counterpart [12]. Moreover, recent studies on the enzymatic hydrolysis of PEF powder highlighted the possibility of both surface functionalization and the polymer recycling process, thus opening up the prospects for a higher-value application of this material [13,14]. 

Today many companies are very interested in developing processes for production of FDCA to be used as a monomer for polyester, polyamide, and polyurethane synthesis. As a result, several patents have been recently published on this subject; among the most recent ones, Dumesic et al. [15] patented the process for FDCA production from C6 sugars, by oxidizing HMF to FDCA with and without separating HMF from the reaction solution containing the by-products. HMF is obtained by dehydrating sugars in a lactone solvent using a Brönsted or Lewis acid catalyst and is oxidised using molecular oxygen and a metal supported catalyst in the absence of a base. For this reason, FDCA is extracted at the end of the reaction using an aromatic solvent. Another patent, published in 2017, converts HMF to FDCA with molecular oxygen using a homogeneous metal salt catalyst and water as solvent [16]. Moreover, FDCA can be produced through an enzymatic pathway starting from glucose or other sugar derivatives; the patented enzymes can perform the desired reaction with high specificity and efficiency [17]. Other two-step processes were patented by Sequeira et al. for the integrated process that generates HMF from aqueous carbohydrate solution and oxidises it into FDCA [18]. Van Harven et al. [19] produced a mixture of 2,4-FDCA and 2,5-FDCA from a disproportionation of furoic acid salts, obtained from furfural oxidation in an alkaline solution and metal-based catalyst. Other patents on the production of purified and dried FDCA using different HMF derivatives as the starting materials have been reported [20,21].

From the industrial standpoint, some pilot scale plants have already been developed [22,23]. Synvina, the joint venture company between BASF and Avantium, has already started a pilot plant for PEF production and is also developing technologies for PEF recycling [24]. Metal-supported catalyst and oxygen are mainly used in these pilot-scale plants. Nevertheless, the precise route for the synthesis of FDCA has not yet been identified, but the current technology for terephthalic acid production using metal/bromide catalysts is being evaluated. One drawback of these catalytic systems is the use of corrosive media and dangerous compounds, which make the process polluting. 

Therefore, the preparation of active and stable metal-supported catalysts, also combing two metals in the form of an alloy, can be of great of interest [25]. The most commonly used monometallic supported catalysts are based on Au [26,27,28,29,30], Pd [2,31,32], and Pt [33,34,35,36,37]. The tailoring of metal particles in terms of size and shape can be a useful tool for increasing catalyst activity and stability. AuCu [38,39,40] and AuPd [5,41,42,43,44,45] bimetallic nanoparticles in the form of alloys and core-shells were found to be active in HMF, showing superior properties compared to their monometallic counterpart. 

However, the support must also be taken into account in a process development, since it is very well known that a catalyst prepared with the same metal active phase may display different catalytic performances depending on the support used [1,46,47,48,49]. Thus, the study of innovative processes aiming at the preparation of metal-supported catalysts, characterized by high surface area and high metal dispersion, is becoming a very important topic. The selection of the method for support preparation is a key factor for catalyst development, since it can affect the thermal and mechanical properties of the material, together with other chemical-physical features such as surface area porosity. As an example, porous ceramics with an open-cell structure are considered suitable catalyst supports; however, some industrial applications might require high temperature-resistant catalysts together with high porosity systems to facilitate mass transfer. Magnesia ceramics prepared with the spray-freeze drying technique made it possible to prepare high-thermal-strength materials, which were also characterized by a large surface area [50]. The spray-freeze drying (SFD) technique is an industrial process which consists of removing water from frozen samples by sublimation and desorption under a vacuum; it has also been applied to the preparation of nanostructured materials because it made it possible to maintain the nanometric size of the phase, while avoiding the agglomeration and segregation of components [51], while at the same time, increasing the stability of the system [52,53]. Moreover, this technique can be used for the homogeneous embedding of active phases into the support, minimizing the possibility of phase separation on a molecular scale. Much effort has been devoted to the formulation of the starting suspension for the identification of the optimal quantity that maximizes stability, safety, and marketability of a given product [54]. 

In this work, special attention was paid to the development of synthetic procedures for the preparation of high-surface-area supports. At first, the spray-freeze drying approach was used for the preparation of round, highly porous TiO_2_-SiO_2_ grains with a size in the 10–100 μm range; then, a microemulsion procedure was optimized to obtain titania of high surface area and small particle size. Pt-based catalysts supported on a nanostructured TiO_2_-SiO_2_ matrix were prepared by SFD. The support was prepared by heterocoagulation of the nanometric suspension of the oxides together with the metallic salt in a self-assembling approach, which exploits the surface charge of different materials to induce the spontaneous organization of the starting materials [55]. The samples obtained from nanosol heterocoagulation were then used to prepare granules using the spray-freeze-granulator, thus leading to the formation of a micrometric catalyst. 

Moreover, the possibility to convert the HMF selectively using a photocatalyst active under sunlight at ambient temperature was investigated. First, the effect of titania preparation was studied, comparing the activity of a TiO_2_ homemade support (TiO_2_-m) prepared by microemulsion and commercial titania (TiO_2_-c). Microemulsion—which is defined as an isotropic and thermodynamically stable dispersion made up of water, oil, and surfactants whose diameters vary from approximately one to 100 nm [56]—has become one of the most-studied methods for the synthesis of nanomaterials [57]. The preparation of nano-oxides by microemulsion is a very interesting field, which has been widely studied in literature [58,59,60,61,62,63,64,65,66]. 

Currently, there is a growing interest in the photocatalytic synthesis of organics, mainly the oxidation of hydroxyl functional groups to aldehydes, because it enables preventing the use of strong chemical oxidants, toxic solvents and by-products, high temperatures, and pressures [67,68,69,70,71,72,73,74,75,76,77].

Therefore, the goal of this paper was to investigate the selective oxidation of HMF by both a heterogeneous batch process and a photocatalytic approach, using Pt- and Au-TiO_2_-based supported catalysts and devoting special attention to material preparation for promoting the catalytic activity and selectivity. 

## 2. Results and Discussion

### 2.1. HMF Oxidation Using Heterogeneous Catalysts

In the present study, Pt/TiO_2_/SiO_2_ samples prepared by the spray-freeze drying technique were tested in the liquid-phase oxidation of HMF in base-free conditions.

#### 2.1.1. Catalyst Preparation and Characterisation

The preliminary approach to the spray-freeze drying technique for catalyst preparation deals with the study of the reagents used as starting materials and the optimization of their amounts to be used in the synthesis. From the industrial standpoint, formulation is a key step in the development of new materials, since the relative amount of the reagents used may affect the chemical-physical properties of the final solid [54].

**Figure 1:**
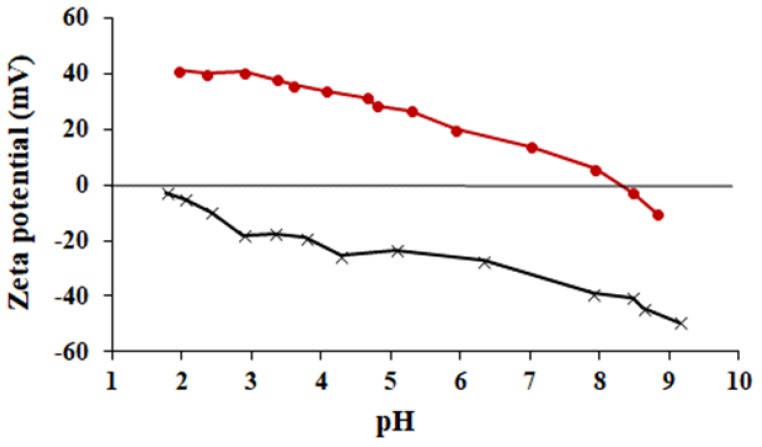
Zeta potential of SiO_2_ (x) and TiO_2_ (•) as a function of pH.

With this aim, the measurements of the zeta potential of colloidal SiO_2_ (LUDOX HS-40) and the water suspension of commercial TiO_2_ (AEROXIDE P25) have been investigated (Figure 1) in a wide range of pH.

**Figure 2:**
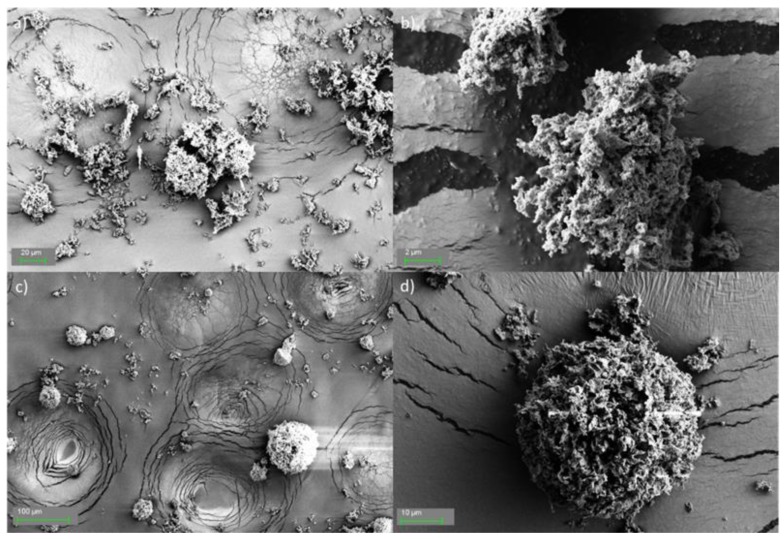
SEM images of the sample TiSi (TiO_2_:SiO_2_ 1:0.5 *w*/*w*) characterized both by irregular shaped grains (**a**,**b**) and spherical granules (**c**,**d**).

The results obtained highlighted the different behaviors of the two materials; titania was characterized by a positive zeta potential in the range two–eight, having its isoelectric point at 8.5, while silica showed a negative zeta potential in the full studied range. Moreover, the very low potential values (between −30 and −50 mV) of SiO_2_ indicated its high colloidal stability. By exploiting the opposite superficial charge, which characterizes these oxides, it is possible to design new materials by heterocoagulation. The spray-freeze drying of the suspension containing both titania and silica in the ratio 1:0.5 (*w*/*w*) made it possible to obtain micrometric round grains as reported in Figure 2. 

**Figure 3:**
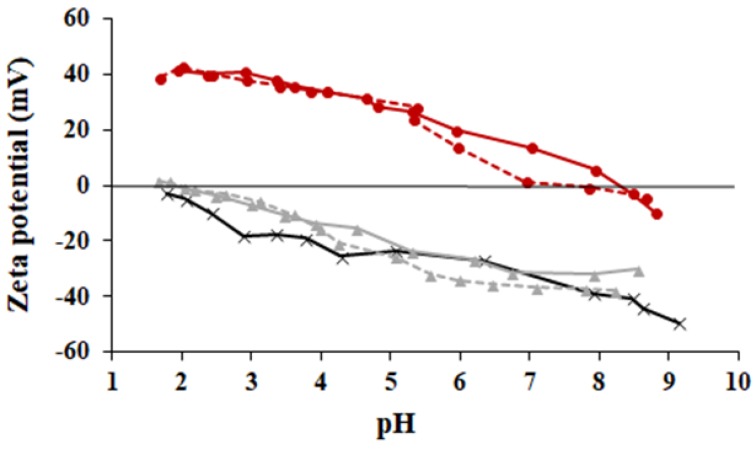
Zeta potential of suspensions containing SiO_2_ (x), TiO_2_ (•, solid line) and Pt containing mixtures—TiPt (•, broken line), TiSi (▲), and TiSiPt (▲, broken line)—as a function of pH.

The use of the same procedure for preparing only TiO_2_-based materials did not permit obtaining regular round granules; furthermore, silica was fundamental in the process of heterocoagulation, since it helped increase the mechanical strength of the grains. Silica also had the role of promoting granulation, i.e., the formation of more spherical and homogeneous granules (Figure 2c,d). Additionally, the addition of a very small amount of SiO_2_ helped in the preparation of these micro-size grains with a packed porosity, even though the obtained samples also contained some irregularly shaped grains (Figure 2a,b), typically obtained in the granulation of TiO_2_. The preparation of a mixed TiO_2_-SiO_2_ material modified the zeta potential. In Figure 3 the measurements of the zeta potential of starting materials are reported and compared to the TiO_2_-SiO_2_ 1.0.5 wt % (TiSi) and platinum-containing catalysts (TiPt, TiSiPt). The isoelectric point of the TiSi sample was 1.9, thus completely changing what was previously observed with starting materials. The presence of silica, although in small amounts, made it possible to reverse the TiO_2_ zeta potential sign, thus demonstrating that TiO_2_ was surrounded by silica NPs. The addition of Pt metal salts was taken into account; (NH_3_)Pt(NO_3_)_2_ was dissolved in the aqueous suspension containing the two oxides before spray-freezing. Pt addition did not lead to any significant change in the zeta potential curve. This trend was also confirmed by the preparation of the TiPt sample. Thus, TiSi and TiSiPt were characterized by very low Z potentials, indicating that the suspension used for spray-freeze drying is stable.

The presence of silica strongly increased the surface area of the material (Table 1). In fact, while silica colloid has a surface area of 210 m^2^/g, titania P25 is characterized by a very low value (46 m^2^/g) and the mixing of the two in the ratio TiO_2_:SiO_2_ 1:0.5 brings the surface area up to 100 m^2^/g. It is important to note that the spray-freeze drying technique, also, is responsible for the increased surface area of the sample, which is an important feature to be taken into account in catalyst preparation. As a matter of fact, the spray-freeze drying of titania P25 suspension led to a significant increase in the surface area of the sample (59 m^2^/g), despite the fact that grain formation did not occur without silica. The addition of platinum reduced the surface area of Ti and TiSi slightly. 

**Figure 4:**
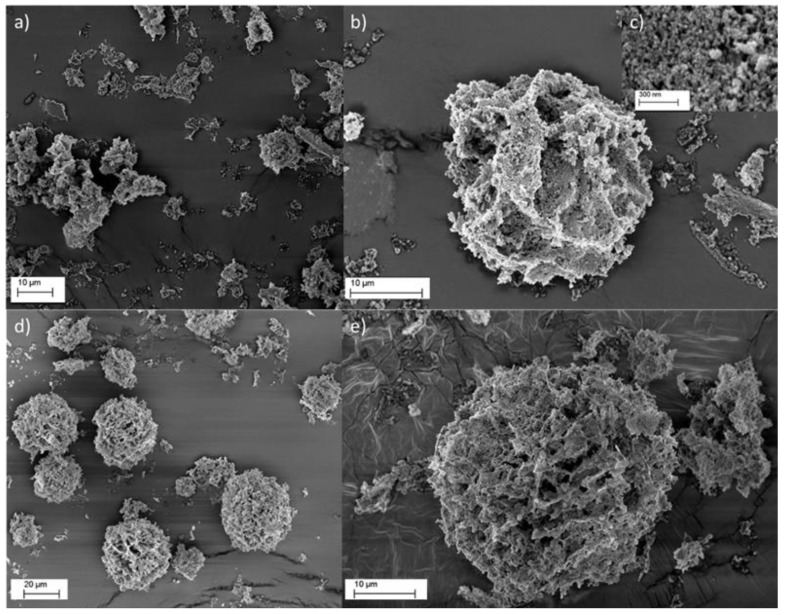
SEM images of (**a**,**b**) TiPt and (**d**,**e**) TiSiPt; (**c**) high magnification SEM image of TiPt sample nanostructuring.

Furthermore, the SEM images in Figure 4 show that grain formation and the macro-porosity of the material are not affected by platinum introduction. However, sample TiPt, which was prepared without the addition of silica, confirmed once again that only the titania suspension did not permit a proper spray-freeze drying process, preventing homogeneous round grain formation (Figure 4a,b). Nevertheless, even in this case, the preservation of the nanostructuring was demonstrated (Figure 4c).

**Figure 5:**
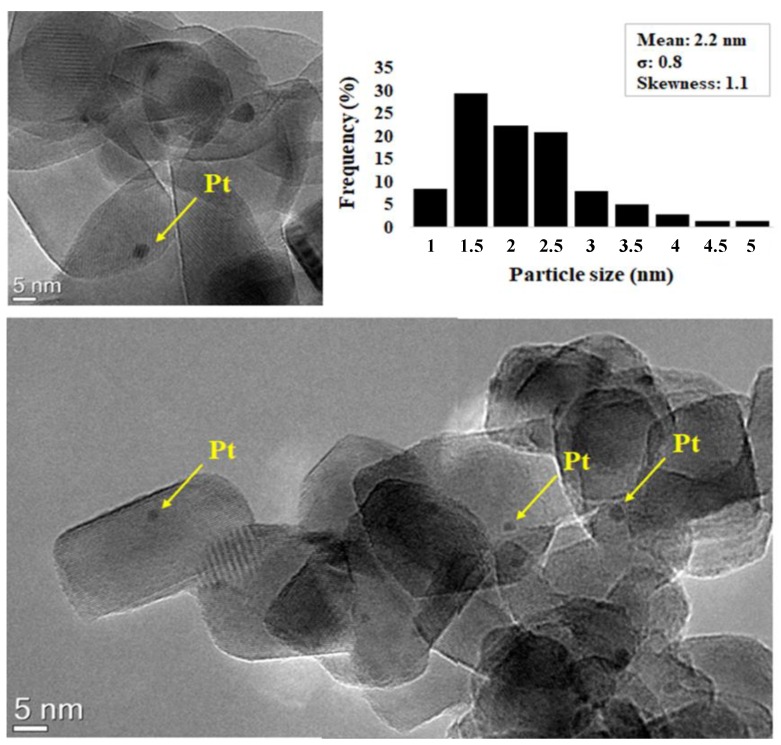
TEM images and TiPt particle size distribution.

**Figure 6:**
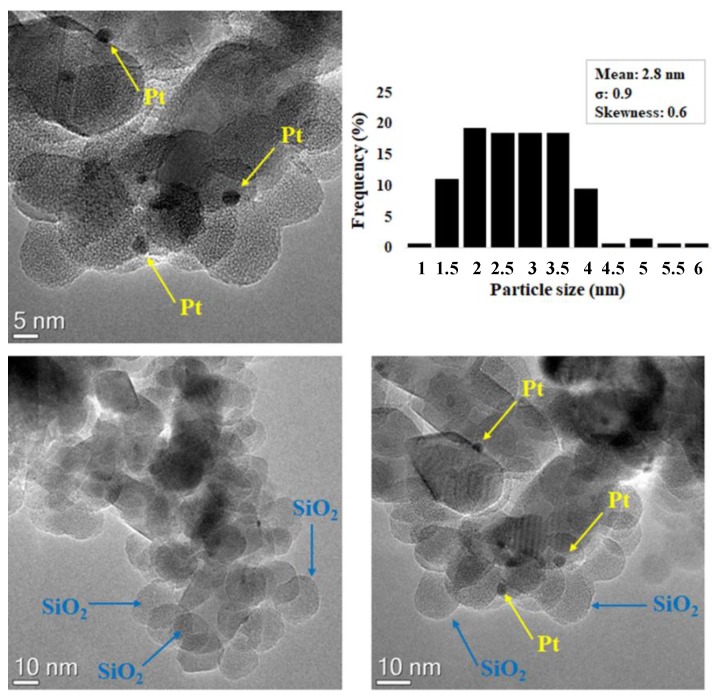
TEM images and TiSiPt particle size distribution.

The prepared samples were treated under H_2_ flow at 400 °C to reduce the metal; then, the particle size distribution was evaluated through TEM analysis. Figure 5 shows TEM images of the TiPt sample showing the presence of small and well-dispersed metal particles with a narrow particle size distribution centered on 2.2 nm. Titania particles can be well distinguished from Pt because of their size and shape. The presence of silica can be seen clearly in the TiSiPt sample (Figure 6) because of its round shape. In this case, also, metal particles are well dispersed and have a very small diameter (2.8 nm), meaning that the presence of silica made it possible to prepare a homogeneous material with dispersed Pt nanoparticles.

#### 2.1.2. Catalytic Tests

**Figure 7:**
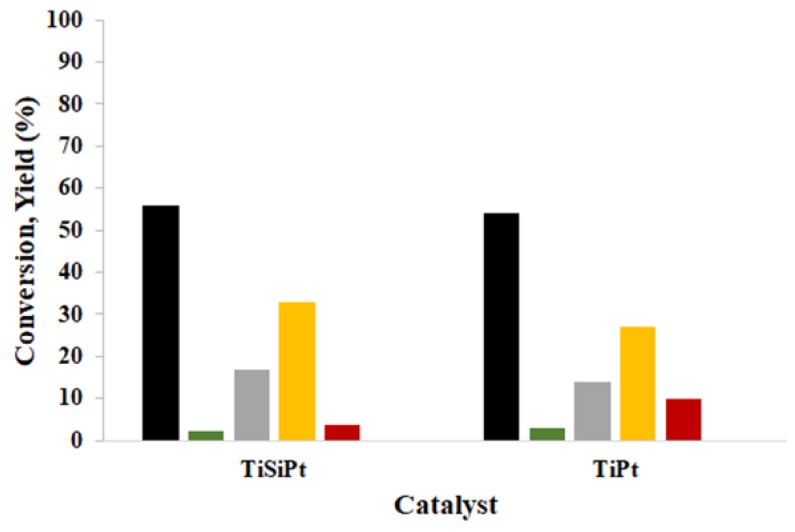
Catalytic activity of the samples TiSiPt, TiPt. Reaction conditions: 6 h, 110 °C, 10 bar O_2_, HMF:Pt 1:0.01, Legend: HMF conversion (■), HMFCA yield (■), DFF yield (■), FFCA yield (■), and FDCA yield (■).

The catalyst TiSiPt and the reference material TiPt were tested in the liquid-phase oxidation of HMF in a batch reactor. The preparation of catalytic materials using the spray-freeze drying process made it possible to obtain TiO_2_-based micrometric grains with a higher surface area compared to the commercial support. Moreover, this synthetic approach permitted the preparation of small Pt nanoparticles characterized by a narrow size distribution and well dispersed throughout the support. All these features favorably affected the catalytic activity; the sample TiPt had an interesting catalytic activity in the absence of a base, leading to more than 50% HMF conversion and 10% FDCA yield. The addition of silica to the system brought a lower FDCA yield (4%), but a higher HMF conversion. In the latter case, the product formed most was FFCA (33%); the lower FDCA yield observed may be related to a different reaction rate of FFCA transformation which, in this case, was the rate-limiting step in the process (Figure 7).

The conduction of the process without a base slowed down the aldehyde oxidation; in fact, in these conditions, HMF conversion is never complete, and both DFF and FFCA are always present in high amounts. Moreover, HMFCA is formed only in traces, meaning that under the studied conditions the oxidation of the alcoholic group is more favored than the transformation of the aldehyde.

**Figure 8:**
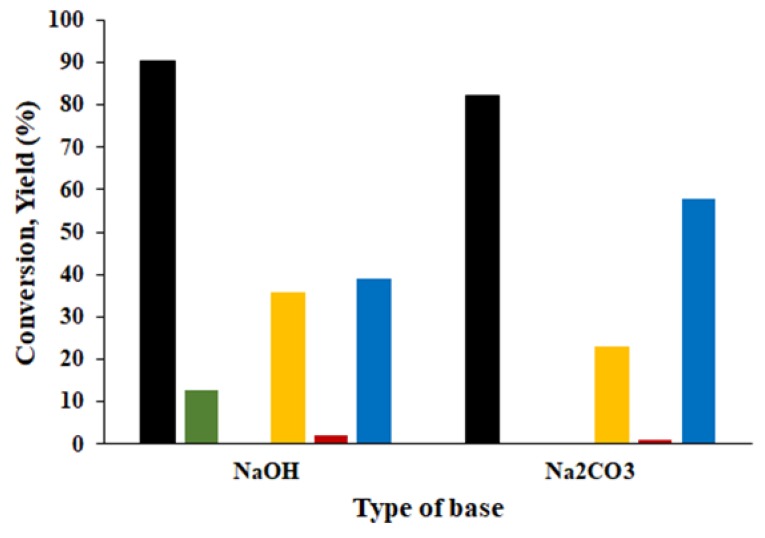
Catalytic activity of the sample TiSiPt with the addition of a different base. Reaction conditions: 6 h, 110 °C, 10 bar O_2_, HMF:Pt:base 1:0.01:2, Legend: HMF conversion (■), HMFCA yield (■), DFF yield (■), FFCA yield (■), FDCA yield (■) and C-loss (■).

Subsequently, a base was added to the system to try to promote the catalytic activity and to check if there was a change in the reaction pathway: results were evaluated after the addition of NaOH and a milder base, such as Na_2_CO_3_ (Figure 8). The reported results highlighted the detrimental effect of the base addition. Surprisingly, the use of a milder base worsened the carbon balance, confirming the role of OH^−^ group in the reaction medium. High pH can cause HMF degradation, but a significant amount of OH^−^ in the solution, in the presence of an active catalyst, can foster HMF oxidation to HMFCA [30]. In both tests, HMF conversion increased, but the side reaction of by-product formation was enhanced. FFCA was observed in high yield, while HMFCA was detected only when sodium hydroxide was used. The basic environment, however, did not stimulate the Cannizzaro reaction on HMF, because no BHMF was observed in the reaction medium.

**Figure 9:**
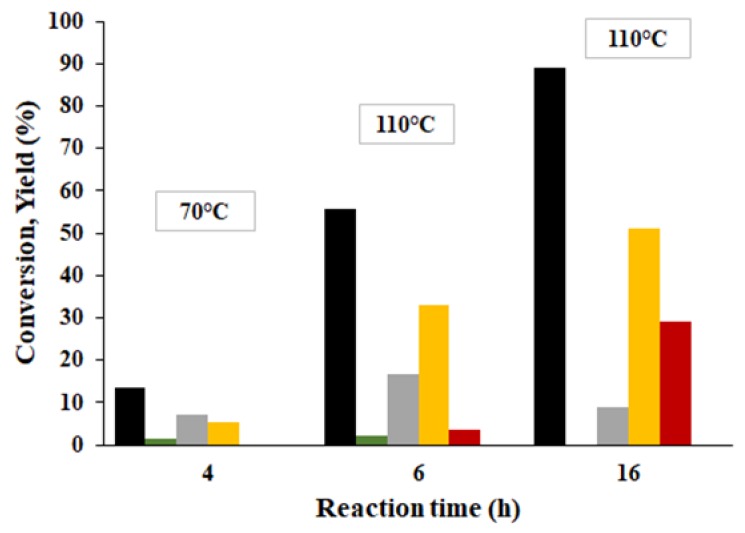
Catalytic activity of the sample TiSiPt at different reaction times and temperatures. Reaction conditions: 10 bar O_2_, HMF:Pt 1:0.01, Legend: HMF conversion (■), HMFCA yield(■), DFF yield(■), FFCA yield(■), and FDCA yield (■).

Lastly, the reaction time and temperature were studied, and the increase of these parameters led to an enhancement of the catalytic performances (Figure 9). In fact, working for 4 h at a mild temperature (70 °C), only 10% HMF conversion was obtained, and DFF and FFCA formed as major products, with a yield of about 5%. The same process conducted at 110 °C for 6h caused a conversion of more than 50%; FFCA was the most important product (33%) followed by DFF (17%). The most interesting result was obtained with a 16h reaction (29% FDCA yield; 89% HMF conversion). FFCA formed in a high amount (51%), while DFF formed with a 9% yield. The absence of HMFCA led to the conclusion that reaction mechanisms passed basically via DFF formation, and without the addition of the base, HMFCA formed only in smaller amounts. Reported results demonstrate once again that when a basic environment is not used, no by-products form.

### 2.2. Oxidation of HMF by a Photocatalytic Process

The second part of the work was devoted to the preparation of a homemade titania (TiO_2_-m), using the microemulsion approach, to be used as the catalyst for the photooxidation of HMF. The effect of base addition, O_2_ content, and the presence of gold were studied. The results obtained were compared with those obtained with commercial TiO_2_ (DT-51 Millennium chemicals) (TiO_2_-c). 

#### 2.2.1. Catalyst Preparation and Characterisation

In order to carry out the photocatalytic oxidation of HMF, some preliminary tests using commercial (TiO_2_-c) and homemade TiO_2_ (TiO_2_-m) were performed. Au nanoparticles were then added to investigate the possibility to induce the selective conversion of HMF. The samples studied are shown in Table 2.

**Figure 10:**
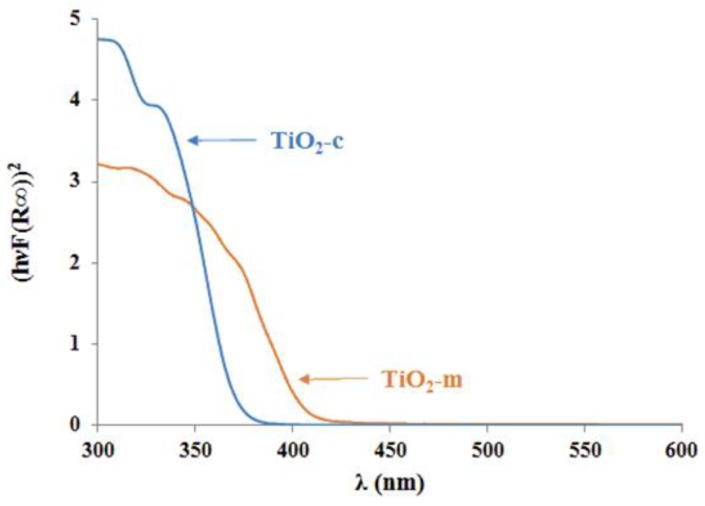
UV-vis diffuse reflectance absorption spectra of TiO_2_-c and TiO_2_-m.

**Figure 11:**
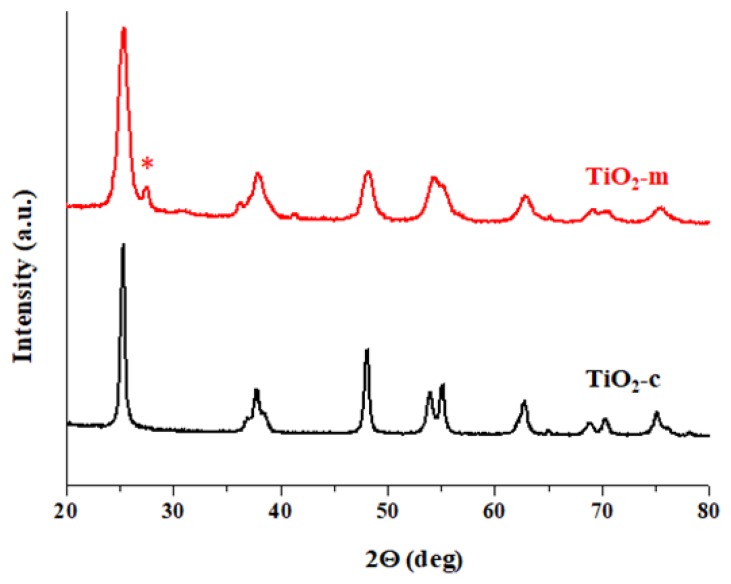
XRD patterns of TiO_2_-c and TiO_2_-m. Rutile phase is assigned as *.

A homemade TiO_2_ support was prepared using the microemulsion method [78] and was compared to the commercial powder. The absorbing capability of photocatalysts in the UV-visible region was measured by UV-Vis diffuse reflectance spectroscopy. The spectra in Figure 10 indicate that absorption of TiO_2_-m is slightly enhanced in the visible region compared to the commercial TiO_2_. This behavior could be mainly due to the presence of a small amount of rutile in the homemade titania TiO_2_-m, as highlighted by the XRD patterns shown in Figure 11. Indeed, it is known that the energy bandgap of rutile is around 3.0 eV, while anatase is characterized by an energy bandgap of 3.3 eV, which is likely the reason for the shift to a visible region for this sample. 

Estimated crystalline sizes of TiO_2_ anatase from XRD analysis are 17 and 8.3 nm for TiO_2_-c and TiO_2_-m, respectively, while TEM images (Figure S1) of both materials confirmed the presence of smaller oxide particles in the homemade oxide. Moreover, BET measurements demonstrated that TiO_2_-m has a larger surface area (Table 2). The results obtained indicated that the procedure used for homemade titania preparation permitted to obtain a photocatalyst with a larger surface area and smaller crystallite size with respect to the commercial powder. 

**Figure 12:**
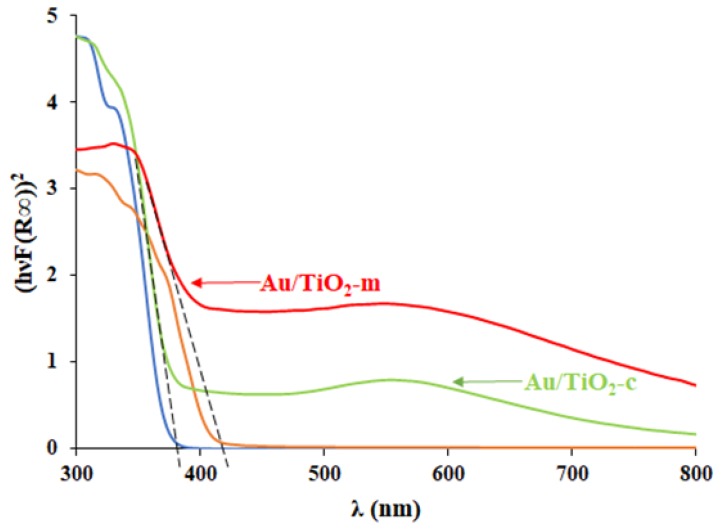
UV-vis diffuse reflectance absorption spectra of Au/TiO_2_-c, Au/TiO_2_-m and corresponding supports.

Au nanoparticles have been introduced by deposition-precipitation both on the commercial and on the homemade TiO_2_, leading to a shift in absorption in the visible range (Figure 12).

**Figure 13:**
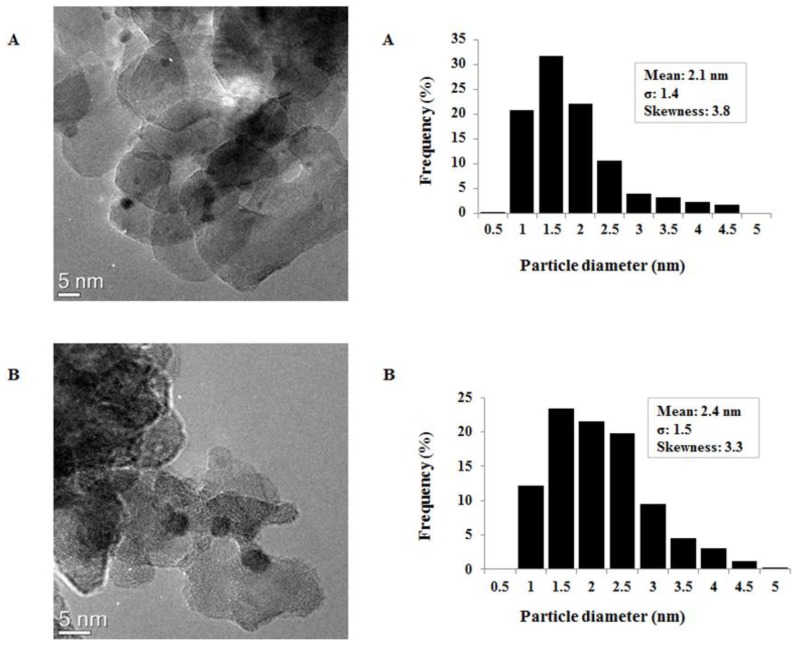
TEM images and particle size distribution of (**A**) Au/TiO_2_-c and (**B**) Au/TiO_2_-m.

Moreover, for metal-containing catalysts a new band located at 550 nm appeared, which is peculiar for this kind of material and is due to plasmon resonance of gold. TEM images of the Au-containing photocatalysts (Figure 13) show that metallic particles are well-dispersed on both TiO_2_ with a size of 2 nm. The larger surface area and smaller titania particles which characterized TiO_2_-m sample, did not significantly influence the particle size and distribution of gold, which resulted to be very similar to what was obtained over commercial TiO_2_. Indeed, in both cases, the deposition precipitation method made it possible to prepare 2 nm Au nanoparticles, homogeneously distributed on the support.

#### 2.2.2. Photocatalytic Tests

**Figure 14:**
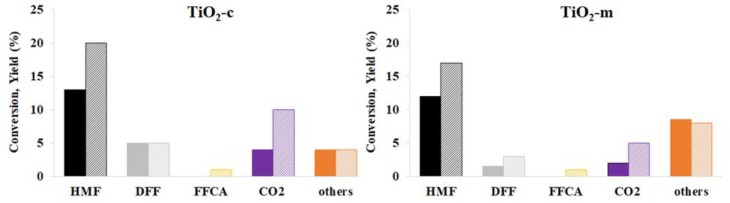
Photocatalytic activity of the commercial (TiO_2_-c and Au-TiO_2_-c) and homemade samples (TiO_2_-m and Au-TiO_2_-m). Reaction conditions: 8 h, 30 °C, HMF 0.08 M. Legend: HMF conversion (■), DFF yield (■), FFCA yield (■), CO_2_ yield (■), and others (■). Bare supports are represented by full bars; gold-supported catalysts have bars with lines.

The materials prepared were tested in the photocatalytic oxidation of HMF in water, using simulated solar radiation. The reaction temperature was rigorously maintained at 30 °C using a water-cooling system. At first, pristine titania (commercial and homemade) were used. The conversions of the two different titania (TiO_2_-c and TiO_2_-m) are very similar (Figure 14), despite the different UV-vis diffuse reflectance absorption spectra measured. The reaction did not occur in the dark, and no oxidation was observed in the absence of catalyst and oxygen. 

**Scheme 2:**
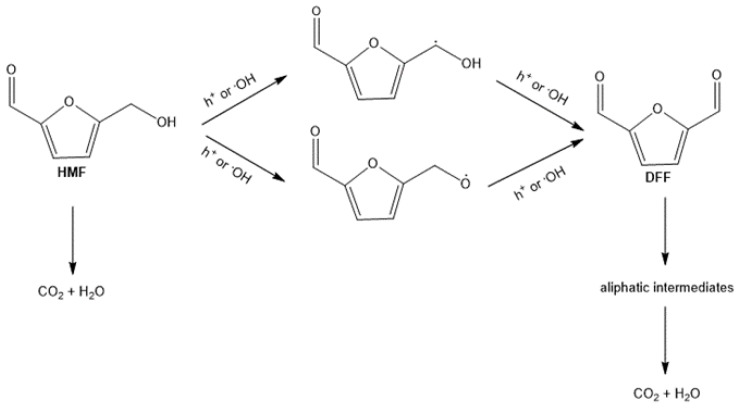
Proposed reaction pathway towards DFF formation over TiO_2_-based samples [67].

The main identified products in the reaction were 2,5-diformylfuran (DFF) and CO_2_. The suggested mechanism for the formation of DFF and CO_2_ can be summarised as reported in Scheme 2, similarly to what has been suggested by Palmisano and co-workers [67]. The reaction starts from the abstraction of hydrogen with respect to an OH-group in HMF molecule by either h+ or ·OH, with subsequent formation of the aldehyde. DFF may then be further oxidized and completely mineralized to CO_2_ and H_2_O through aliphatic intermediates: something that actually explains the presence of carbon dioxide and other ring-opened by-products as observed at low retention times from HPLC analysis (Figure S2). 

As a matter of fact, both HPLC and ESI-mass analysis showed the presence of other compounds, within which we saw the presence of maleic acid. This compound has already been found in literature as a derivative of HMF oxidation [79] using H_2_O_2_ [80,81]; so it is possible that hydrogen peroxide formed by radical reaction using TiO_2_ as the photocatalyst in water [69] promoted the formation of this compound. Other by-products might be formed in reactions with reactive oxygen species produced by the photocatalytic reaction over TiO_2_ (such as h+, ^•^O_2_^−^, ^•^OH, and H_2_O_2_); in fact, it is well known that aliphatic intermediates can be obtained from molecules that are able to form a peroxy-bridge, as also observed in the photooxidation of phenol [69]. 

When comparing the commercial to the homemade titania, DFF and CO_2_ yield was lower for TiO_2_-m, but a higher carbon loss was observed using this material. This seems to indicate that the homemade titania has a higher reactivity in the formation of by-products. Indeed, the HMF conversion value was very similar to that obtained with commercial titania. 

An effective improvement of the photocatalytic activities was observed with the introduction of gold onto the catalyst. Nevertheless, when increasing the HMF conversion, a significant enhancement of CO_2_ yield was observed, suggesting the presence of a metal causing mineralization reactions [82]. Indeed, DFF selectivity was higher using bare TiO_2_, suggesting that the observed high activity of metal-containing systems is mainly associated with unselective reactions on both the commercial and the homemade supports. Worthy of note was the FFCA formation when Au nanoparticles were deposited on TiO_2_ (Figure 14). 

**The effect of the base on the photocatalytic reaction**

With the aim of improving the selectivity, Na_2_CO_3_ was added in the molar ratio of HMF:Na_2_CO_3_ = 2, to avoid unselective oxidation, since this base was reported to act as a ·OH radical scavenger [72].

**Figure 15:**
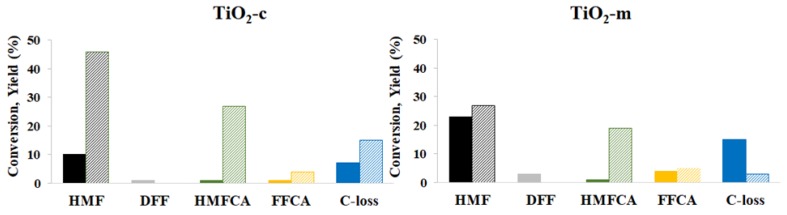
Photocatalytic activity of the commercial (TiO_2_-c and Au-TiO_2_-c) and homemade samples (TiO_2_-m and Au-TiO_2_-m) in presence of a base with HMF:Na_2_CO_3_ = 2 molar ratio. Reaction conditions: 8 h, 30 °C, HMF 0.08 M. Legend: HMF conversion (■), DFF yield (■), HMFCA yield (■), FFCA yield (■), and C-loss (■). Bare supports are represented by solid bars; supported catalysts have lighter, lined bars.

The general effect observed after the base addition is the increase in HMF conversion and also the production of a higher amount of oxidation products, such as FFCA and other by-products; HMFCA appeared with all the catalytic systems, but its formation was deeply enhanced with gold-supported catalysts. The presence of HMFCA is mainly attributed to the presence of a base. Therefore, an interesting consideration can be made by evaluating the results obtained for the two different titanium oxides: HMF oxidation, which passes mainly via DFF formation with the consecutive formation of FFCA, can go through the other pathway in basic conditions, which includes HMFCA formation. (Figure 15). The changed reaction mechanism can be observed even in the case of metal-supported catalysts; in the presence of gold, this effect is even more emphasized where a yield of more than 25% of HMFCA is achieved with Au/TiO_2_-c. Meanwhile, no DFF formation was observed in both cases, indicating that—in the presence of a base—gold is very active in HMF oxidation, while the reaction pathway passes through HMFCA formation, since alcohol oxidation is the rate-limiting step of the reaction.

Moreover, despite the low temperature used in these tests (30 °C), the basic pH enhances HMF degradation, leading to the formation of high-molecular-weight molecules such as humins [11], defined as C-loss. As a matter of fact, other products such as maleic acid were not observed in these conditions. 

**Scheme 3:**
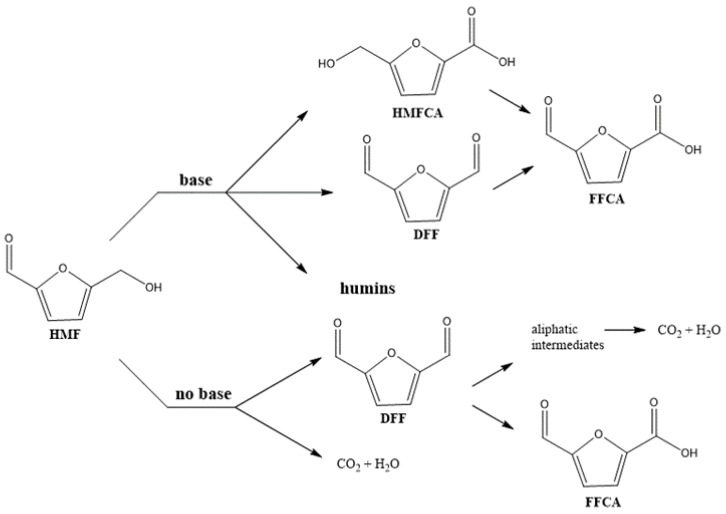
Reaction pathway of HMF photooxidation in the presence and absence of a base.

Scheme 3 sums up the two different pathways of HMF oxidation that are observed for Au/TiO_2_ catalysts and pristine supports. In the presence of a base, the reaction pathway is mainly towards HMFCA and FFCA formation. The results of HMF photooxidation for bare TiO_2_ also showed DFF formation, thus suggesting that FFCA can also be formed via the fast oxidation of DFF using gold-containing systems. The presence of colored, high-molecular-weight products in the reaction mixture confirms the formation of humin-like compounds at basic pH. On the contrary, in the absence of a base, HMF is mainly converted to DFF, which can either be transformed into FFCA using gold-supported catalysts or go through a ring-opening reaction leading to the formation of aliphatic products and CO_2_. Some mineralization products can also be derived from HMF, as reported by Yurdakal et al. [67].

**The effect of O_2_ on the photocatalytic reaction**

The effect of O_2_ on the photocatalytic reaction was investigated using pristine and gold-containing TiO_2_-m, with the aim of elucidating the role of an oxidant during photooxidation (Table 3). Using bare TiO_2_-m, the conversion of HMF was increased from 10% to 22%, leading to a higher formation of DFF, CO_2_, and aliphatic by-products, when a greater amount of oxygen was introduced into the reactor. On the other hand, similar conversions were obtained using the gold-containing catalyst (Au/TiO_2_-m). These results suggest that molecular oxygen plays a pivotal role when bare titania is used as the catalyst. Indeed, it can be activated by the photogenerated electrons and participates in oxidation processes. The presence of O_2_ is also important since it acts as an electron scavenger and reduces the recombination of electrons and holes [83]. The amount of oxygen, however, does not seem to be crucial for the reaction when gold is added to TiO_2_, probably because, in this case, the rate-limiting step is the charge transfer between TiO_2_ and Au, while oxygen activation on a gold surface is not dependent on its concentration [84].

**Figure 16:**
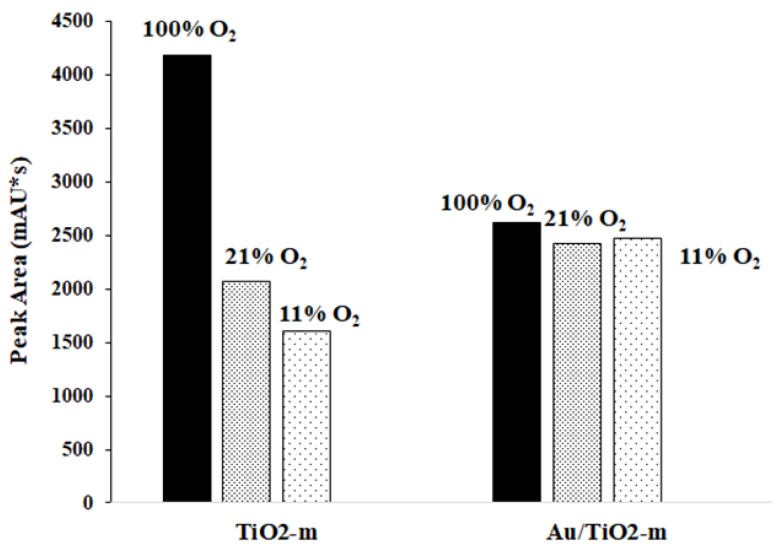
Peak area from HPLC analysis of the aliphatic product obtained at different oxygen contents with TiO_2_-m and Au-TiO_2_-m. Reaction conditions: 8 h, 30 °C, HMF 0.08 M.

The influence of oxygen content on aliphatic product formation was also studied. In particular, the presence of one compound that had a retention time of 12 min (see S2 for the HPLC chromatogram) was noticed and its peak area was considered. The correlation of the peak area obtained with homemade titania catalysts and different amounts of O_2_ is shown in Figure 16. The results obtained highlighted that O_2_ content encouraged the formation of the aliphatic product, especially when the bare support was used. 

These results merely give an overview of the reaction mechanism for HMF photooxidation, which is influenced by the base content, oxygen amount, and presence of the metal. However, in order to find out which radical is actually involved in DFF formation using the prepared systems, some additional tests are currently ongoing, similar to what has been recently reported in literature [25,34,35].

## 3. Materials and Methods

### 3.1. Catalyst Preparation

#### 3.1.1. Preparation of Pt/SiO_2_/TiO_2_ Catalysts by Colloidal Heterocoagulation and Spray-Freeze Drying

Nanostructured micrometric powders made up of silica, titania, and platinum were produced in different compositions using a colloidal heterocoagulation method combined with spray-freeze drying. Multicomponent TiO_2_/SiO_2_ samples were obtained by heterocoagulation, mixing the stable suspension of the two oxides. Titania P25 Areoxide was suspended in acidic water (pH = 3.5). Then colloidal SiO_2_ nanosol (LudoxHS-40, Grace Davison, Columbia, MD, USA) was added after dilution in distilled water to 20 wt %, obtaining a 13 wt % total solid concentration for the preparation of a TiO_2_/SiO_2_ ratio of 1:0.5. The two oxide mixtures were prepared by dripping under stirring 50 g of SiO_2_ Ludox HS in 50 g of TiO_2_P25 (3 wt %). The pH of the silica commercial nanosol was modified up to a value of 4, by means of a cationic exchange resin (DOWEX 50 × 8). This procedure entails the slow addition of the resin under stirring, until pH 4 is reached. The heterocoagulation is promoted at pH 4, corresponding to a slight colloidal destabilization, in order to enhance electrostatic interactions between positively (TiO_2_) and negatively (SiO_2_) charged surfaces. The mixture thus prepared was ball-milled for 24 h using ZrO_2_ balls (5 mm diameter). The Pt precursor (NH_3_)_4_Pt(NO_3_)_2_ was added before and after SiO_2_ addition in order to evaluate any possible differences in physicochemical properties and catalytic performances. The spray-freeze-granulation technique was applied here to obtain micrometric powders starting from nanosols using the lab-scale granulator instrument, LS-2. The suspension was atomized by means of a peristaltic pump, blowing nitrogen gas at 0.4 bar through a 100 μm nozzle and nebulized into a stirred solution of liquid nitrogen, thus enabling an instantaneous freezing of each generated drop. The drops thus frozen were placed into a freeze-drying apparatus with a pressure of 0.15 mbar and a temperature of −1 °C, thus promoting the sublimation process which was completed in 48 h, producing a highly porous granulated powder.

All the granulated samples were then submitted to a thermal treatment (H_2_ flow at 400 °C, 10 °C/min, then isothermal step for 30 min), which was aimed at consolidating the granule structure and reducing any Pt. 

#### 3.1.2. Titania Microemulsion Preparation (TiO_2_-m)

In the photocatalytic study, two types of TiO_2_ were used: Commercial anatase powder DT-51 Millennium chemicals (denominated TiO_2_-c), and a homemade one which was synthetized by using a microemulsion-mediated system (denominated TiO_2_-m) [78]. 

#### 3.1.3. Synthesis of Au/TiO_2_ by the Deposition-Precipitation Method

During the synthesis, a 0.001 M solution of HAuCl_4_·3H_2_O was used at pH 9, adjusted by adding dropwise a 0.1 M NaOH solution. Similarly, the pH of an aqueous suspension (300 mL) of 2 g of TiO_2_ was adjusted to 9. Then a solution of HAuCl_4_·3H_2_O was added dropwise to the TiO_2_ suspension under vigorous stirring at room temperature, maintaining pH 9. Once the entire gold solution was transferred to TiO_2_ suspension, the temperature was increased to 65 °C. Once the temperature reached the desired value, the stirring was continued for 2 h.

As was reported [30], this synthesis is supposed to result in the selective deposition of Au(OH)_3_ on the TiO_2_. The solid was then separated from the solution by centrifugation, washed several times with water to remove chloride, dried at 110 °C overnight, and calcined at 300 °C for 2 h (rate 2 °C/min).

### 3.2. Catalyst Characterisation

Zeta potential measurements on the colloidal samples were performed at 25 °C with the Electrophoretic Light Scattering (ELS) technique by means of the instrument Zetasizer nano ZSP (Malvern Instruments, Malvern, UK). The Smoluchowski equation was applied to convert the electrophoretic mobility to zeta potential. The instrument is equipped with an autotriation which enables the identification of the isoelectric point (IEP) and adds up automatically to the sample KOH 0.1 M or HCl 0.1 M, in order to explore the zeta potential trend within a selected pH range. Measurements were performed on samples diluted at 0.1 wt %. 

The granulated catalysts were observed by scanning electron microscopy using a Field Emission Scanning Electron Microscope, FESEM (Carl Zeiss Sigma NTS, Oberkochen, Germany). Granules were fixed to aluminum stubs with conductive adhesive tape. The elemental analysis was performed by image analysis using FESEM coupled to an energy dispersive X-ray micro-analyzer (EDS, mod. INCA).

The synthesized materials were also examined by high resolution transmission electron microscopy (HR-TEM), using a TEM/STEM FEI TECNAI F20 (ThermoFisher, Hillsboro, OR, USA), which uses a high-angle annular dark field (HAADF) imaging mode at 200 kV. Samples were dispersed on a holey carbon film supported on a copper grid.

XRD was measured at room temperature with a Bragg/Brentano diffractometer (X’pertPro PANalytical) equipped with a fast X’Celerator detector, using a Cu anode as the X-ray source (Kα, λ = 1.5418 Å). Diffractograms were recorded in the range 5–80°2θ, counting for 15 s every 0.05°2θ step.

Catalyst surface areas were measured by a N_2_ physisorption apparatus (Sorpty 1750 CE instruments) and single-point BET analysis methods, in which samples were pre-treated under a vacuum at 120 °C. 

Solid UV-VIS analyses were recorded in a Perkin Elmer Lambda 19 instrument (Perkin Elmer Waltham, MA, USA) equipped with an integrating sphere in the range 280–800 nm. 

### 3.3. Catalytic Tests

Batch reactor: 5-hydroxymehtyfurfural (HMF) oxidation: HMF was oxidised in a 100-mL autoclave reactor (Parr Instrument, Moline, IL, USA) equipped with a mechanical stirrer (0–600 rpm) and tools for the measurement of temperature and pressure. The reactor was filled with 25 mL of distilled water, the appropriate amount of 5-hydroxymethylfufural, NaOH, or Na_2_CO_3_ (2 eq. when specified), and catalyst (HMF/metal molar ratio = 100). The autoclave was purged 3 times with O_2_ (5 bar) and then pressurised at 10 bar. If not otherwise indicated, the temperature was set from 70 to 110 °C, and the reaction mixture was stirred at 400 rpm for 4–6 h. At the end of the reaction, the reactor was cooled down to room temperature, and the solution was filtered, diluted 5 times, and analyzed by an Agilent Infinity 1200 liquid chromatograph equipped with a Aminex HPX 87-H 300 mm 7.8 mm column using a 0.005 M H_2_SO_4_ solution as the mobile phase. The identification of compounds was achieved by calibration using reference commercial samples.

Photoreactor: The essential part of the experimental set-up was performed by the solar simulator that consists of a 300 W Xe-lamp, which generates light in the 250–2500 nm range with an output of 1 sun at a distance of 18 cm. The reactive mixture is placed in a glass photoreactor which in turn comprises the circulating bath, inlet and outlet for gaseous compounds, outlet for sampling liquid products, stirring system, and quartz disk for maintaining light transmission, once the reactor is sealed. Catalysts were then tested for 8 h with 0.08 M aqueous solution of HMF with an HMF/Au molar ratio of 100. The same amount of powder was introduced with the bare TiO_2_. The reaction took place at 30 °C with the irradiance output of 1000 W/m^2^ and spectral range of 250–2500 nm. 

After reaction, samples were separated from the photocatalyst by centrifugation or filtration, diluted by 5 times in deionized water and analyzed by HPLC as described above. 

Gaseous products were analyzed in an off-line Thermo Focus GC with a carbon molecular sieve column (CARBOSPHERE 80/100 6*1/8) and TCD detector.

## 4. Conclusions

The oxidation and photooxidation of HMF in water can provide environment-friendly methods to produce DFF and FDCA, an important molecule serving as the starting point for the synthesis of bio-polymers. Catalytic materials based on TiO_2_ were shown to be active in both the thermal and photoactivated conditions, and the reaction network was significantly affected by the catalyst method of synthesis and composition. Micro-sized TiO_2_-SiO_2_ grains with packed porosity and high surface area, containing very small and well dispersed Pt nanoparticles (2 nm), were active in the liquid-phase oxidation of HMF at neutral pH, forming DFF, FFCA, and FDCA as the most important products. The addition of a base led to a change in the reaction mechanism, forming HMFCA as the first intermediate; however, a large amount of high-molecular weight by-products was present because of HMF degradation in these high pH conditions.

Microemulsion was used to prepare homemade TiO_2_ having a higher surface area and smaller crystallite size compared to commercial DT-51. The use of both materials for HMF photooxidation led to the production of DFF and CO_2_ as the main products; moreover, some aliphatic intermediates, such as maleic acid, were observed. The different chemical-physical properties of the two oxides did not seem to have a strong influence on the selectivity of the process in the conditions studied. 

The introduction of gold enhanced the activity but also decreased the DFF selectivity, while molecular oxygen was shown to play a pivotal role, especially when bare titania was used as the catalyst.

## Figures and Tables

**Table 1:** Silica, Platinum content, and surface area of the samples prepared.

Sample Name	TiO_2_:SiO_2_ (*w*/*w*%)	Pt (wt %)	Surface Area (m^2^/g)
SiO_2_	-	-	210
TiO_2_ (P25)	-	-	46
Ti	-	-	59
TiSi	1:0.5	-	100
TiPt	-	0.5	41
TiSiPt	1:0.5	0.5	91

**Table 2:** Prepared samples and main characterization data obtained from XRD, UV-vis spectroscopy, and BET measurements.

Sample	Crystallite Size/nm		λ/nm	S_BET_/m^2^/g
	TiO_2_ Crystallites	Au Crystallites		
TiO_2_-**c**	17	-	371	82
TiO_2_-**m**	8.3	-	413	132
Au/TiO_2_-**c**	17	<3	380	89
Au/TiO_2_-**m**	8.3	- *	429	130

* Au XRD peaks could not be seen by XRD due to the presence of rutile.

**Table 3:** Photocatalytic results for TiO_2_-m and Au-TiO_2_-m with different oxygen contents. Reaction conditions: 8 h, 30 °C, HMF 0.08 M.

Catalyst	O_2_ Content (vol %)	HMF Conv. (%)	DFF Yield (%)	FFCA Yield (%)	CO_2_ Yield (%)	Others Yield (%)
**TiO_2_-m**	11	10	2	-	3	5
	21	12	2	-	2	8
	100	22	5	-	8	9
**Au-TiO_2_-m**	11	12	2	-	2	8
	21	17	3	1	5	8
	100	15	3	1	8	3

## Supplementary Materials

The following are available online. Figure S1. TEM images of the studied titania support. TiO2-m (microemulsion) and TiO2-c (commercial); Figure S2. HPLC analysis of the reaction mixture TiO2-m (microemulsion) and TiO2-c (commercial); Figure S3. Schematic representation of solar simulator. Table S1. Solar simulator and reactor technical parameters.
