# Supplementary material for: Selective Oxidation of HMF via Catalytic and Photocatalytic Processes Using Metal-Supported Catalysts
Source: Molecules. 2018 Oct 27;23(11):2792. doi: 10.3390/molecules23112792 (PMC6278393; doi:10.3390/molecules23112792)
Supplement: Supplementary file 1 [file molecules-23-02792-s001.pdf]

Selective oxidation of HMF by heterogeneous and photocatalytic processes using metal supported catalysts

Alice Lolli[a] , Valeriia Maslova[a,b], Danilo Bonincontro[a,b], Francesco Basile[a], Simona Ortelli[c], Stefania Albonetti[a,b,*]

a Dip. Chimica Industriale "Toso Montanari", Università di Bologna, Viale Risorgimento 4, 40136 Bologna (BO), Italy

b Université de Lyon; C2P2 - UMR 5265 (CNRS - Université de Lyon 1 - CPE Lyon), Équipe Chimie Organométallique de Surface CPE Lyon, 43 Boulevard du 11 Novembre 1918, FR-69616 Villeurbanne Cedex. France

c ISTEC-CNR, Institute of Science and Technology for Ceramics, National Research Council, Via Granarolo 64, 48018, Faenza, Italy

* Corresponding authors: stefania.albonetti@unibo.it

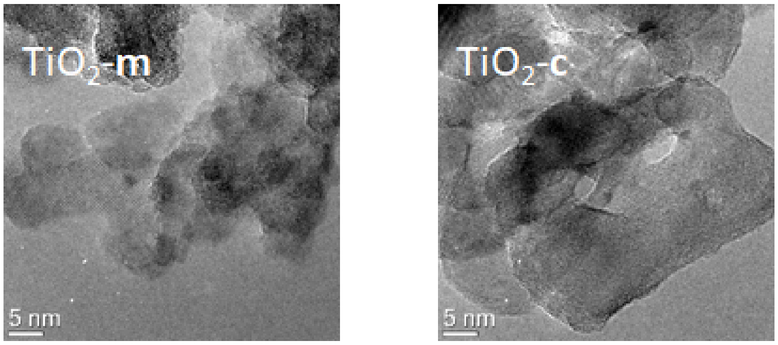

Figure S1. TEM images of the studied titania support. TiO_2-m (microemulsion) and TiO_2-c (commercial).

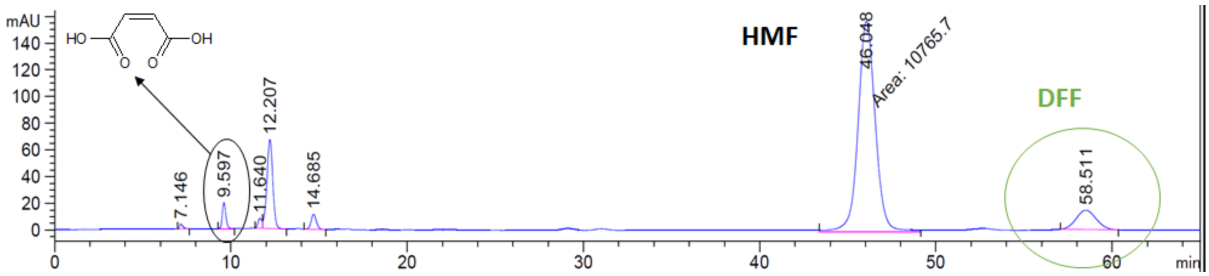

Figure S2. HPLC analysis of the reaction mixture TiO_2-m (microemulsion) and TiO_2-c (commercial) catalysts.

Table S1. Solar simulator and reactor technical parameters.

**Solar simulator**	
Producer	LOT-QuantumDesign
Solar simulator	LS0306
Power supply	LSN254
Lamp type	300 W xenon short arc
Spectral range	250-2500 nm
Illumination diameter	40 mm
Irradiance	1 sun @ 180 mm
**Home-made glass Reactor**	
Material	Glass
Type	Batch
Total volume	217 cm^3
Diameter	46 mm
Components	Jacket of circulating cooling bath, inlet and outlet, quartz disk on the top for maintenance of light transmission

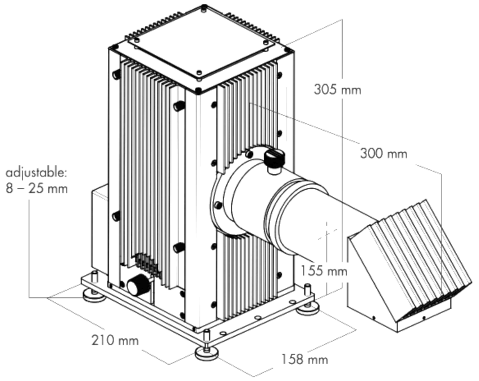

Figure S3. Schematic representation of solar simulator.
